# Mining multi-site clinical data to develop machine learning MRI biomarkers: application to neonatal hypoxic ischemic encephalopathy

**DOI:** 10.1186/s12967-019-2119-5

**Published:** 2019-11-21

**Authors:** Rebecca J. Weiss, Sara V. Bates, Ya’nan Song, Yue Zhang, Emily M. Herzberg, Yih-Chieh Chen, Maryann Gong, Isabel Chien, Lily Zhang, Shawn N. Murphy, Randy L. Gollub, P. Ellen Grant, Yangming Ou

**Affiliations:** 1 Division of Newborn Medicine, Department of Pediatrics, Massachusetts General Hospital, Harvard Medical School, Boston, MA 02114 USA; 2 Fetal Neonatal Neuroimaging and Developmental Science Center (FNNDSC), Boston Children’s Hospital, Harvard Medical School, 401 Park Drive, Landmark Center 7022, Boston, MA 02115 USA; 3 Computer Science & Artificial Intelligence Lab (CSAIL), Massachusetts Institute of Technology, Cambridge, MA 02139 USA; 4 Laboratory of Computer Science, Massachusetts General Hospital, Harvard Medical School, Boston, MA 02114 USA; 5 Department of Psychiatry and Department of Radiology, Massachusetts General Hospital, Harvard Medical School, Boston, MA 02114 USA; 6 Neuroradiology Division, Department of Radiology, Boston Children’s Hospital, Harvard Medical School, Boston, MA 02115 USA; 7 Computational Health Informatics Program (CHIP), Boston Children’s Hospital, Harvard Medical School, Boston, MA 02115 USA

**Keywords:** Neonatal encephalopathy, Hypoxic ischemic encephalopathy, MRI, Biomarkers, Machine learning, Outcome prediction, Bioinformatics

## Abstract

**Background:**

Secondary and retrospective use of hospital-hosted clinical data provides a time- and cost-efficient alternative to prospective clinical trials for biomarker development. This study aims to create a retrospective clinical dataset of Magnetic Resonance Images (MRI) and clinical records of neonatal hypoxic ischemic encephalopathy (HIE), from which clinically-relevant analytic algorithms can be developed for MRI-based HIE lesion detection and outcome prediction.

**Methods:**

This retrospective study will use clinical registries and big data informatics tools to build a multi-site dataset that contains structural and diffusion MRI, clinical information including hospital course, short-term outcomes (during infancy), and long-term outcomes (~ 2 years of age) for at least 300 patients from multiple hospitals.

**Discussion:**

Within machine learning frameworks, we will test whether the quantified deviation from our recently-developed normative brain atlases can detect abnormal regions and predict outcomes for individual patients as accurately as, or even more accurately, than human experts.

*Trial Registration* Not applicable. This study protocol mines existing clinical data thus does not meet the ICMJE definition of a clinical trial that requires registration

## Background

**Fig. 1:**
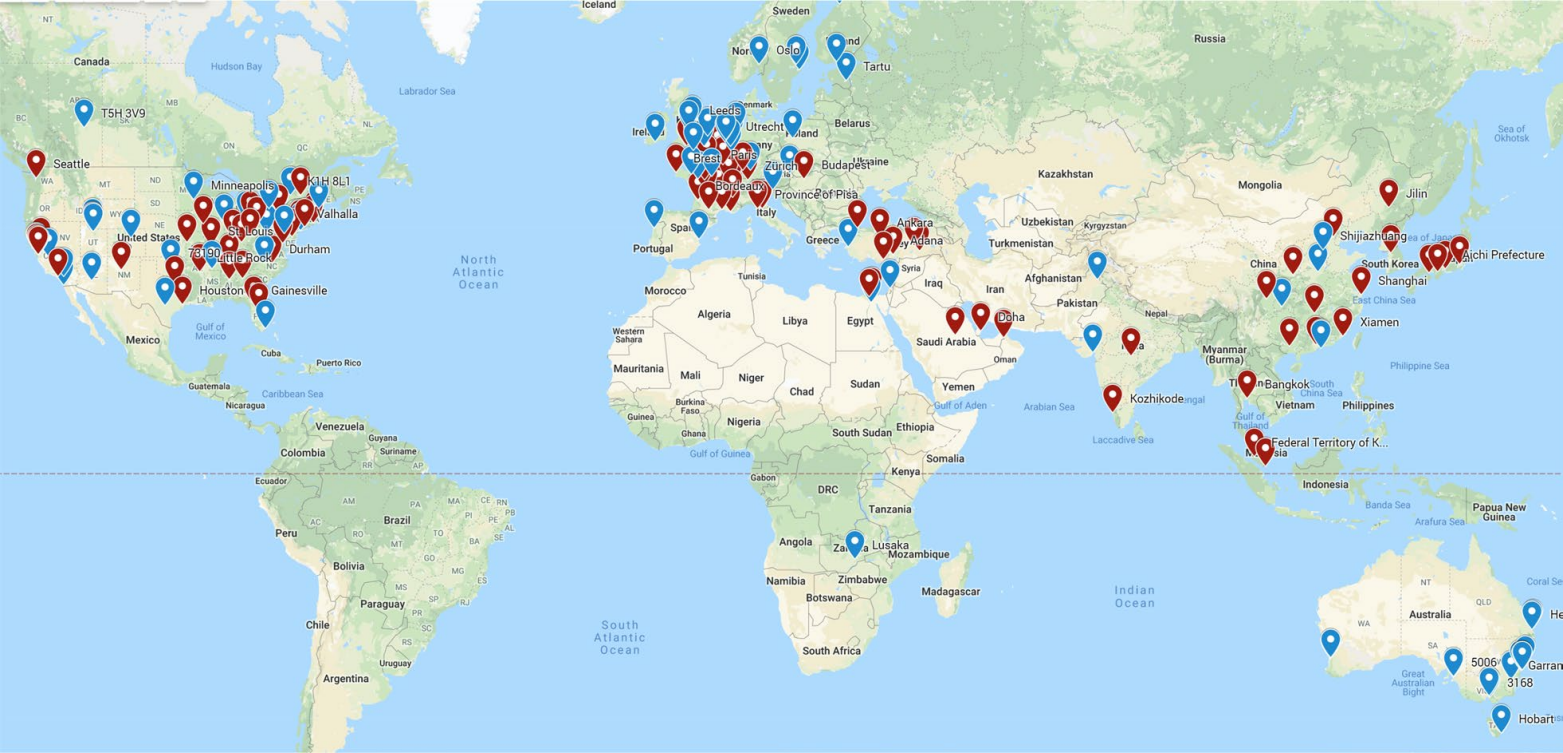
Need for MRI in HIE-related clinical trials. Each icon notes a hospital/site where at least one HIE-related clinical trial is ongoing. Red icons are hospitals that use MRI and blue icons are those that do not use MRI in their trials. Among 108 ongoing clinical trials pertaining to HIE at hospitals from 33 countries in 5 continents, roughly half of the hospitals use MRI as part of their trials, highlighting the widespread need for MRI biomarkers that can detect HIE lesions at infancy and predict HIE outcomes at 2 years of age. This figure was created based on searching the key word “Hypoxic Ischemic Encephalopathy” in the public website for clinical trial registries (https://clinicaltrials.gov). The search was in June 2019. We manually added each site in all 108 resulting HIE trials on the Google My Map website

Hypoxic ischemic encephalopathy (HIE) affects 1–5/1000 of live births, and is a leading cause of morbidity and mortality in childhood [1, 2]. Although the implementation of therapeutic hypothermia (TH) reduces infant mortality and chronic disability (by 2 years of age) [3–5], neurodevelopmental impairments are still common in survivors [6–8]. Specific impairments vary across surviving patients, motivating the development of prognostic biomarkers. There is progress in developing clinical [9–11], biochemical [9–12], and serum [12, 13] biomarkers. However, it remains unclear whether or not MRI can serve as a non-invasive and highly sensitive biomarker to improve outcome prediction in the early postnatal period [14–16]. Indeed, in the 108 clinical trials that are ongoing for HIE worldwide [17] (Fig. 1), MRI is used in over half of them to assess all stages of HIE management including: diagnosis, prevention, prognosis, intervention, and rehabilitation.

Currently, expert scoring of neonatal MRI is used in clinical trials to predict 2-year outcomes [18–23], but limitations exist. *First*, experts score MRI by looking for lesion presence or absence in selected key brain regions (e.g., thalamus, basal ganglia, etc.), and by looking at whether HIE lesions are unilateral or bilateral, locally confined or globally distributed, etc. Different scoring systems range from 6 score levels [19] to recently 57 score levels [20]. As scoring criteria get more complex, expert scoring takes more time, requires more training and becomes more uncertain (20–40% intra-/inter-reader variability [15]). *Second*, sensitivity can vary in multi-site data, with a detailed scoring system developed in 2018 reporting a 92.3% sensitivity in one cohort but 42.1% in another cohort [20]. *Third and more importantly*, up to 50% of HIE patients have MRIs visually interpreted as normal by experts [24, 25], despite 5–8% of them having adverse outcomes at 18–22 months [4, 11, 26]. This remains unexplained to date. To address these three limitations in expert MRI scoring systems, our goal is to develop quantitative, objective and machine learning-powered algorithms and software to detect HIE lesions during neonatal stages and predict 2-year HIE outcomes.

Machine learning (ML) approaches identify lesions, extract MRI injury features, and find the feature subset (i.e., patterns) that best predicts outcomes [27, 28]. Accuracy is measured by comparing the predicted outcome (assuming unknown during prediction) with the actually known outcome [29]. The promise has been shown in brain tumors [30–32], Alzheimer’s Disease (AD) [33–36], neuroscience [37, 38], stroke [39], epilepsy [40], psychiatric disorders [41–43], traumatic brain injury (TBI) [44, 45], pediatric brain tumor [46], etc. HIE poses 3 unique challenges to ML: (i) lack of data: public data exists for hundreds of patients with brain tumors [47, 48], AD [49, 50], etc.; however, annotated MRIs with linked clinical and outcome data are rare in HIE and none exists publicly. (ii) unique difficulty in lesion detection: radiologists look for regions of abnormal signals in T1-/T2-weighted MRI, diffusion-weighted image (DWI) and Apparent Diffusion Coefficient (ADC) maps [19, 51]. However, HIE-induced T1, T2, DWI and ADC changes rapidly evolve and are entangled with rapid normal neonatal brain development [51–53]. This is not an issue in mature brains; (iii) normal MRI but adverse outcome: 20–50% HIE patients have normal MRI (i.e., no lesion detected visually) [24, 25], but 5–8% of them still develop adverse outcomes [3, 11, 26]. This cannot be explained by expert scores (standard for HIE) or current ML methods (designed for other diseases), which require explicit lesion detection [28, 29].

Our study has three novelties to address these three challenges. *First*, we will retrospectively collect multi-site clinical data that can be used to develop MRI analysis tools. Clinical data includes demographics, hospital assessments, treatment, MRI, as well as outcomes at neonatal intensive care unit (NICU) discharge and outcomes at 2 years. The secondary use of hospital-hosted clinical data has received increasing attention with value added clinical results [54–56]. We plan to use registry- and informatics-driven approaches to retrospectively pull and regularly update data from NICU and hospital-hosted clinical archives at Massachusetts General Hospital (MGH) and Boston Children’s Hospital (BCH). This is different from many clinical trials, which require significant funding and a pre-set timeframe (often years) to prospectively collect patient data. In contrast, we anticipate a relatively low cost and shorter time frame for data collection; the data will be continuously evolving as new patients or visits are added. Moreover, starting with actual clinical data will facilitate the translation to clinical practice. We present our planned efforts in data collection and MRI analysis algorithm design. Special emphasis is placed on addressing the quality of the clinical data, especially multi-site data. *Second*, we propose a new ML framework specific for HIE lesion detection, where the uniqueness is to disentangle HIE-induced and normal-development-related ADC signals in ML. The specific hypothesis we plan to test is that the quantitative deviation from normative neonatal brain Apparent Diffusion Coefficient (ADC) atlases, which we developed recently [57], can facilitate quantitative and automated HIE lesion detection and outcome prediction at an accuracy comparable or higher than experts. We will use ADC maps derived from diffusion tensor MRI [58, 59], as they are commonly used to identify HIE lesions clinically in the first week of age [24, 60]. Radiologists identify lesions by searching for regions of abnormally low ADC values corresponding to decreased water diffusion [24, 60]. However, the normal ranges of ADC variations vary in space (different brain regions) and in time (as the brain develops rapidly in infancy), making expert interpretation error-prone [15, 61]. We recently developed the first-of-its-kind normative ADC atlases, which quantified the normal range of ADC variations in space and in time [57] (see Figs. 4 and 5). Based on this, we can objectively quantify the deviation of a patient’s ADC values from normal variations at every voxel (i.e., 3D pixel) in the brain [62]. Deep learning lesion detection frameworks still apply. But, instead of using voxels from training patients that ignored the normal variations in their spatiotemporal locations, which is not a problem in mature brains, we will feed new channels (we term Z_ADC_ map) to specifically separate HIE-related ADC changes from spatiotemporal ADC changes from normal neonatal brain development. *Third*, other than current radiomics approaches that mostly rely on explicit lesion detection, we will develop a novel “radiomics without lesion detection” approach, which relies on regional and tract-wise MRI features throughout the brain to address the unique issue in HIE that some neonates with clinically-normal MRI (no detectable lesions) may still develop adverse 2-year outcome.

## Methods and design

### Overview

**Fig. 2:**
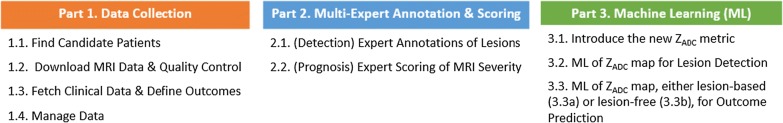
Overview of the three key pillars of our study

This study is approved by the Institutional Review Board at MGH and BCH. Figure 2 outlines the three key components in our study.

### Part 1. Data collection

Figure 2 (Part 1) shows the major steps in data collection.

#### Part1.1. Find candidate patients

We will use two sources to find candidate patients. Our primary source is the NICU registry. The NICU registry contains patient’s diagnosis, medical record numbers (MRNs), and demographic information (birth weight, gestational age, etc.). We will query the NICU registries for patients who

- were term born (> 36 weeks gestation);
- had a clinical diagnosis of HIE and were free of other major neurological disorders.

A second source is the hospital-wide database. In the big data era, an increasing number of hospitals around the world have informatics tools that allow authorized personnel to search patients by diagnosis, or by the International Classification of Diseases (ICD) codes [63–65]. From our registry data, we identified a list of ICD codes commonly used for HIE (Table 1). We will use these ICD codes to search patients that are not captured in the registry.

**Table 1:** ICD codes for HIE as a secondary source to query candidate patients

ICD-9	Meaning	ICD-10	Meaning
*768.70*	Hypoxic-ischemic encephalopathy, unspecified	*P91.60*	Hypoxic ischemic encephalopathy [HIE], unspecified
*768.71*	Mild hypoxic-ischemic encephalopathy	*P91.61*	Mild hypoxic ischemic encephalopathy [HIE]
*768.72*	Moderate hypoxic-ischemic encephalopathy	*P91.62*	Moderate hypoxic ischemic encephalopathy [HIE]
*768.73*	Severe hypoxic-ischemic encephalopathy	*P91.63*	Severe hypoxic ischemic encephalopathy [HIE]
*779.2*	Cerebral depression, coma, and other abnormal cerebral signs in fetus or newborn	*P91.4*	Neonatal cerebral depression

Italic plain font for ICD-9 (left half of the table) and italic font for ICD-10 codes (right half of the table)

#### Part1.2. Download MRI data and quality control

We will use medical record numbers (MRNs) of candidate patients to search and download their MRI data. MGH’s mi2b2 workbench [66] allows authorized users to find and copy DICOM-format MRIs from the Radiology archives to a local cache. BCH’s ChRIS platform offers the same function [103, 104]. From the candidate patients identified in the previous step, we will include those with:

- both structural and diffusion MRIs. At BCH and MGH, the T1-weighted MPRAGE sequence typically has a 1 mm isotropic high resolution. Diffusion MRI typically has a 2 mm isotropic resolution, with at least 6 (often 20–35) gradient directions at b value 1000 s/mm^2^.
- reasonable image quality (e.g., no severe motion or artifacts in either MRI sequences), as visually reviewed by a trained assistant.

#### Part1.3. Fetch clinical data, define outcomes

##### Clinical variables

We will include the following maternal variables: maternal demographics, parity, significant medical history, prescription medications during pregnancy, alcohol/tobacco/elicit substance use, mode of delivery, complications around delivery (e.g. chorioamnionitis, prolonged 2nd stage of labor), or a sentinel event (e.g. fetal bradycardia, uterine rupture, umbilical cord prolapse), and placental pathology, if available. We will also include the following infant data: anthropometric measurements, APGAR scores, umbilical cord gas and/or the infant’s initial blood gas (if available), medications administered during the initial hospitalization, the presence of clinical or electrographic seizures, mode of feeding at hospital discharge, abnormalities on the discharge physical examination, length of stay, and discharge disposition (e.g. deceased, home, transferred). A trained expert will obtain this information from the electronic health records (EHRs). Absence of explicit information will not be used as a negative finding.

##### Outcomes

We will also retrieve from EHR:

- outcome at NICU discharge: deceased or survival;
- outcomes at 2 years of age: neurocognition at 18–24 months, including both continuously-valued developmental assessments and categorical outcomes, as listed in Table 2.

**Table 2:** Definition of long-term neurocognitive outcomes at ~ 2 years of age

i. Continuously-valued outcome	Numerical domain scores
BSID-III (Bayley Scale of Infant Development, Version III)	Cognitive (ranging 50–150) Language (ranging 50–150) Motor (ranging 50–150)

ii. Binary-valued outcome	Definition
Developmental delay	YES (1) if BSID-III ≤ 85 in any domain OR any documentation by a physician that there is a significant delay in motor, cognitive, or language development that requires intervention; otherwise NO (0)
Cerebral palsy	As documented in the EHR by the medical provider
Motor impairment	YES (1) if BSID-III ≤ 85 in the motor domain OR any noted motor abnormality documented in the EHR by a medical provider; or NO (0)
Visual/hearing impairment	As documented in the EHR by a medical provider

#### Part1.4. Manage data

We will use REDCap [67], a HIPAA-compliant, secure, and user-friendly web application, to facilitate manual entry of clinical variables from the EHR and expert review. Entries into REDCap will be reviewed by collaborating neonatologists.

MRI data will be anonymized, stored, and analyzed using HIPAA-compliant computers and high-performance computer clusters as provided by MGH and BCH.

### Part 2. Multi-expert annotation and scoring

Expert opinions will serve as references to validate the proposed MRI analysis tools (Fig. 2, Part 2).

#### Part 2.1. Annotations of lesions

We will use expert consensus as the Ref. [68], since postmortem histologic specimens are rarely available. We plan to have 3 experts (> 5 years of clinical pediatric neuroradiology experience) independently annotate HIE lesions on each ADC map. While there may be uncertainties among experts [15, 61], the consensus will be more accurate compared to individual expert annotation. We define consensus as found by the STAPLE tool, which, loosely speaking, is an improvement of majority voting by computing a probabilistic estimate of the true regional annotation from multiple experts’ annotations [69]. Given that up to 50% of HIE patients may not have clinically-detectable lesions in the neonatal MRI, an expert will leave a blank annotation if he/she does not find lesions in a patient.

#### Part 2.2. Expert scores to predict outcomes

Predicting outcomes requires a different approach. The “ground truth” is available from the clinical records. We will use at least 2 experts to score the severity of the neonatal MRI and to test whether the proposed algorithms/tools outperform expert scores in predicting the “ground-truth” outcomes. The expert scores will be based on the NICHD–NRN scoring criteria [19] (National Institute of Children Health and Human Development, Neonatal Research Network), as listed in Table 3.

**Table 3:** The NICHD–NRN scoring system [19]

NICHD–NRN Scores, 2012 [19]	Criteria
0	Normal
1A	Minimal cerebral lesions only, without basal ganglia thalamus (BGT), anterior limb of internal capsule (ALIC), posterior limb of internal capsule (PLIC) or watershed (WS) infarction
1B	More extensive cerebral lesions, without BGT, ALIC, PLIC or WS infarction
2A	Any BGT, ALIC, PLIC or WS infarction without any other cerebral lesions
2B	Either BGT, ALIC, PLIC or WS infarction AND any other cerebral lesions
3	Hemispheric devastation

### Part 3. Developing machine learning algorithms/tools for lesion detection and outcome prediction

**Fig. 3:**
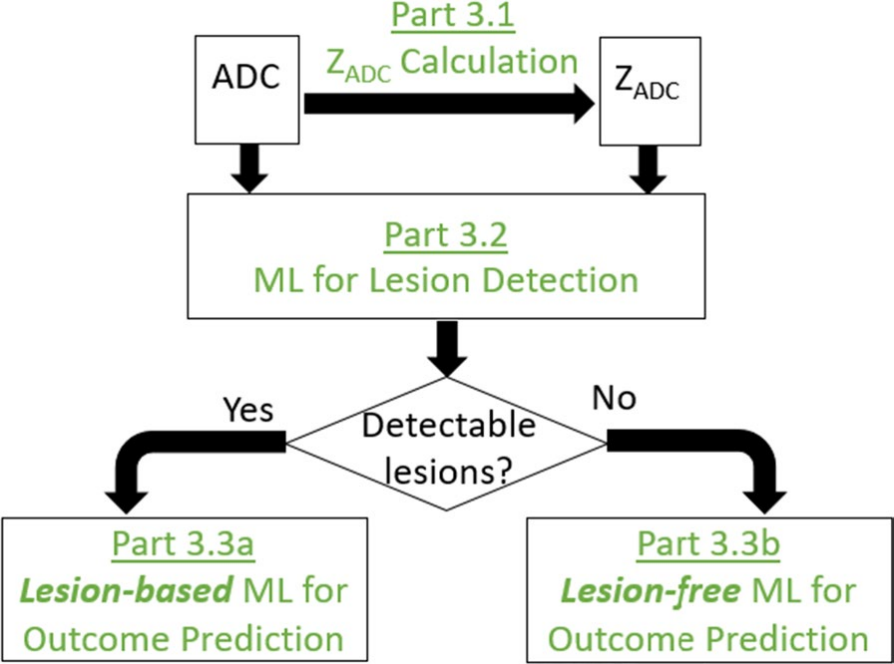
Flowchart for ML-driven lesion detection and outcome prediction

Figure 3 shows the flowchart of Part 3. We start from feature extraction (Part 3.1, Z_ADC_ calculation). The raw ADC map and the novel Z_ADC_ feature map will be fed to ML-based lesion detection (Part 3.2). Depending on whether there are detectable lesions in the patient, ML-driven outcome prediction (Part 3.3) will either go through lesion-based outcome prediction, which will use ADC, Z_ADC_ and detected lesions as input (Part 3.3a), or go through lesion-free outcome prediction, which will use ADC and Z_ADC_ for outcome prediction (Part 3.3b).

#### Part3.1. Feature extraction—introducing the Z_ADC_ measurement

**Fig. 4:**
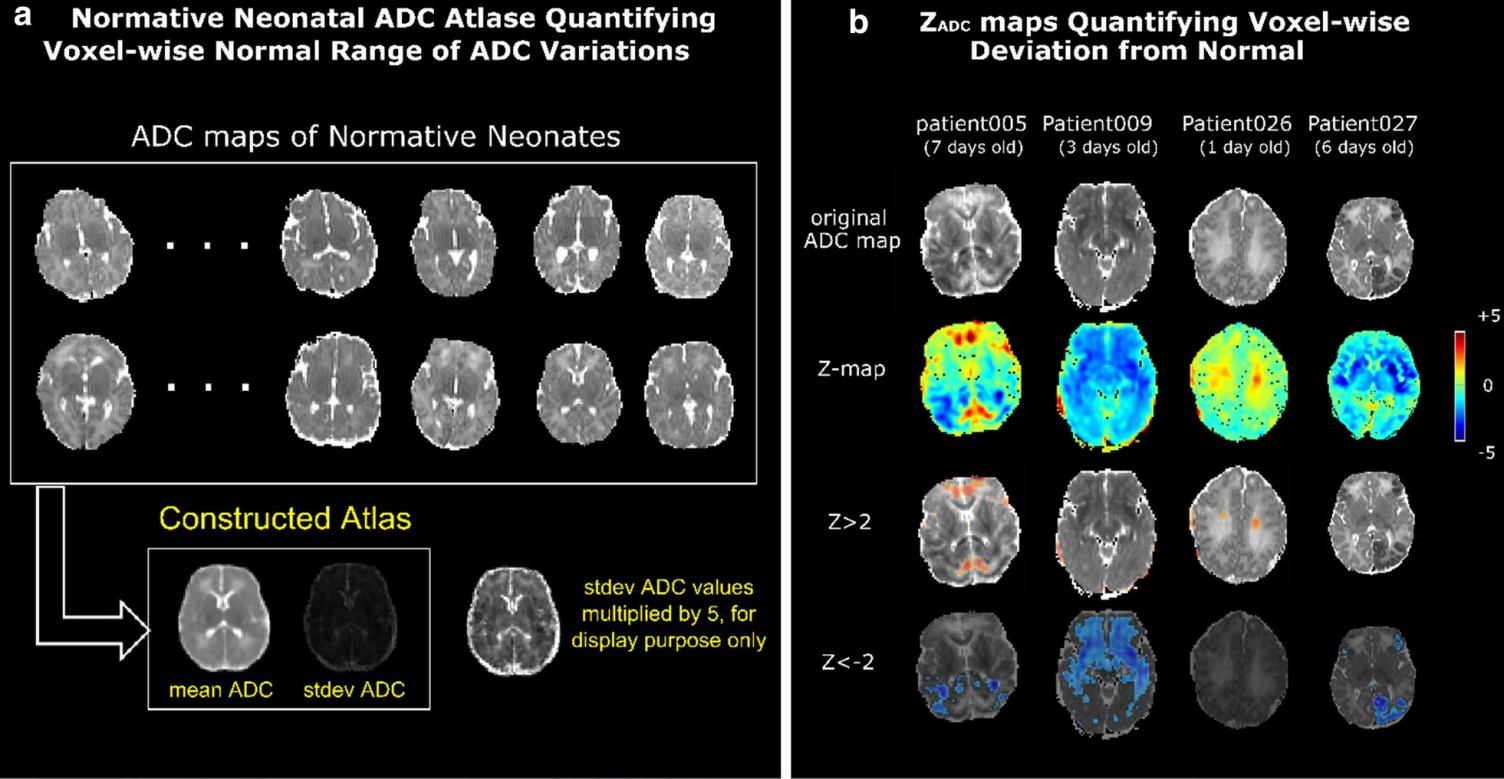
Z_ADC_ map as a new MRI measurement to quantify the voxel-wise deviation from normal. **a** From ADC maps of normative neonates (left, upper part), we constructed the mean ADC (left) and standard deviation (stdev) of the ADC map (left, lower part). **b** Demonstration of Z_ADC_ maps in four neonates with HIE. The top image of each column is a representative axial ADC map through areas of injury. The color coding indicates the Z-score relative to the age matched normative atlas in **a**. This approach allows us to detect regions of decreased ADC, which have been associated with outcome, as well as explore the relevance of high ADC values, which occur with vasogenic edema

Uncertainty in the visual interpretation of neonatal brain ADC maps arises for a number of reasons including the rapidity of brain development during infancy (i.e., temporal uncertainty) and the variation of normal ADC values across different brain regions (i.e., spatial uncertainty). Our recently developed normative ADC atlases quantified the mean and standard deviation (stdev) ADC values at every voxel in the brain in a normative cohort of 13 term-born neonates who were scanned in the first 2 weeks, with a median age of 4 days at the time of MRI scan (see Fig. 4a) [57]. This allows us to quantitatively compare a patient’s ADC value y(u) at a voxel u (first row of panel b) to the mean μ(v) and standard deviation σ(v) ADC values at the anatomically-corresponding location v on the atlas (Fig. 4a). Here the correspondence will be found by the extensively-validated [70] patient-to-atlas DRAMMS deformable registration [71]. This will convert a patient’s ADC value into a Z_ADC_ value, i.e., Z_ADC_(u) = [y(u) − μ(v)]/σ(v), for each voxel u in the patient space (second row of panel 7b). The Z_ADC_ value quantifies the deviation from normal at every voxel in a patient [62].

Our pilot results in 8 patients showed that regions with Z_ADC_ < − 2 (i.e., last row in Fig. 4b) identified HIE lesions at an accuracy comparable to consensus of time consuming expert labelling (between experts Dice overlap at 71% and the average algorithm-and-expert Dice overlap at 69%) [72, 73]. The next steps will include testing the effects of the novel Z_ADC_ map for lesion detection in larger cohorts and in data from more institutions, as enabled by the planned dataset. Finding spatial lesions patterns informative of outcomes, and eventually, patient-centered outcome prediction is the goal (Fig. 4). Machine learning (ML) is well suited for these tasks.

#### Part3.2. Machine learning of Z_ADC_ and ADC for automated lesion detection

##### Preprocessing

We will convert series of 2D DICOM files into an integral 3D NIfTI file for each MR sequence. We will anonymize the patient name and the date of birth in the NIfTI header. We will perform N4 to correct for the inhomogeneity of MRI signals caused by inhomogeneity of the magnetic field in the scanner [74], we will do field-of-view normalization to make sure brain MRIs are of the same scope (from brain stem to the top of the brain, excluding any neck, shoulder or even upper chest that were included in the raw scan) [75], we will skull strip the brain MRI to keep only the brain and remove the eyes, faces, skull, etc. [76, 77]; we will do automated segmentation [78] on the MPRAGE structural image to parcellate the brain into 62 anatomic structures, and we will non-rigidly map the segmented anatomic regions to the diffusion MRI space [71] so that diffusion MRI including the ADC maps will also be segmented into structures.

##### Question 1: Can Z_ADC_ detect HIE lesions more accurately than single experts?

**Fig. 5:**
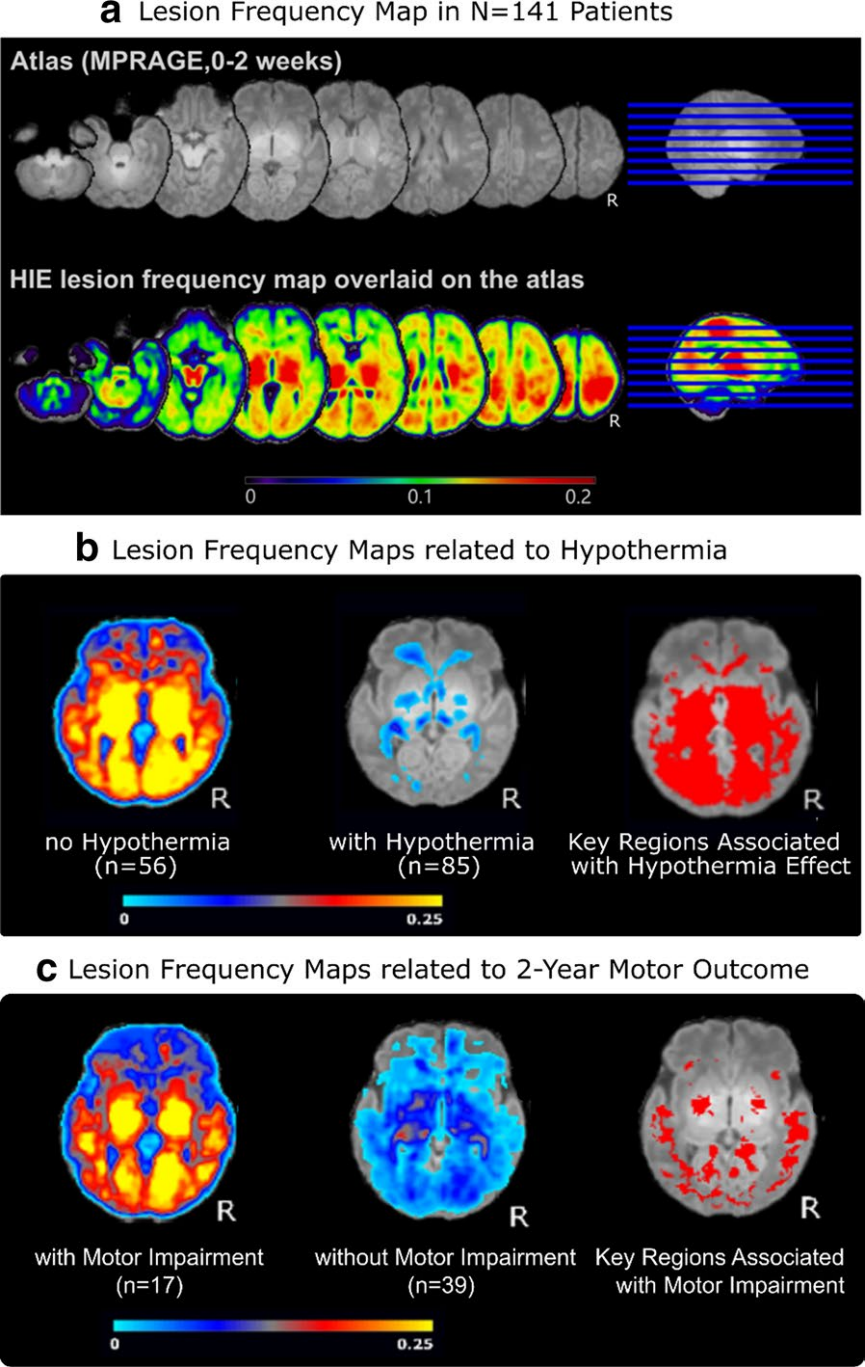
Probabilistic lesion frequency atlases to quantify key brain regions associated with treatment and outcome. **a** Lesion atlas in 141 patients; **b** lesions atlases in patients having not undergone therapeutic hypothermia (left) and having undergone therapeutic hypothermia (middle), and the brain regions that show significant decreases in lesion frequency with treatment (right); **c** lesion atlases in patients with (left) and without (middle) motor impairment at ~ 2 years, and the regions that were more often injured with this outcome. In the second row of **a** and first two columns in **b** and **c**, the color at a voxel denotes the frequency of lesions (i.e., percentage of patients in our cohort having lesions at this voxel), which is indexed by the color bar at the bottom of each panel. In the right column of **b** and **c**, the red color shows the voxels where the two sub-cohorts in the left and middle columns of each panel have significant differences in lesion occurrence. That is, in **b**, the red in the right column shows the regions where patients having received hypothermia have significantly lower frequencies of lesions than patients not undergoing hypothermia

The calculation of Z_ADC_ map is essentially a feature calculation or feature extraction step, which we consider as part of machine learning in this paper. We will test the accuracy of atlas-based lesion detection by the sensitivity, specificity and Dice overlap between computer-generated and expert consensus labelled lesion regions [72, 73]. One way of using our novel Z_ADC_ feature map is to simply threshold Z_ADC_ values at each voxel (Fig. 4). This is analogous to a Bayesian classifier—from normal controls we have built a Gaussian model of normal distributions of ADC values at each voxel, and given a new patient’s ADC value at this value, Z_ADC_ is related to the likelihood of this patient’s this voxel being lesioned or not. We will first test simply thresholding the Z_ADC_ map at various threshold values (e.g., − 1, − 1.5, − 2, − 2.5). Another way of using the novel Z_ADC_ map is to use more complex machine classifiers to identify whether every voxel in the brain is affected by HIE lesions (e.g., voxel-wise normal-vs-lesion machine classification), given the ADC and Z_ADC_ values at this voxel and in the geometric neighborhood of this voxel. We will test whether deep learning classifiers [79, 80] (e.g., 2D and 3D U-Net [81], V-Net [82]), which is free of hand-crafted features and characterizes each voxel by its multi-scale neighborhood information, offer additional advantages over classic classifiers that rely on hand-crafted features of a voxel (e.g., Support Vector Machine [30], and Random Forest [68]). We will test the effect of using the Z_ADC_ value at each voxel as inputs with and without the ADC values of the voxel, and quantify whether this improves lesion detection accuracy compared with using the ADC values alone [68]. We also plan to test whether post-processing based on prior knowledge can further improve the accuracy of lesion segmentation. One post-processing can be the opening (i.e., dilation) and/or closing (i.e., erosion) morphological operations. Another post-processing is to regularize the computer-detected regions with voxel-wise probability of lesion occurrence in HIE populations (see Question 4 and Fig. 5a). Frameworks for lesion-atlas guided/regularized lesion detection can be used [83].

We will use Dice overlap and receiver-operating-curves (ROCs) to quantify the accuracy with regard to expert consensus labelled lesion regions. We will compare our accuracy with the literature [68] and with multiple experts. We will conclude that our algorithm is more accurate than single experts if it achieves a higher Dice overlap with expert consensus than the Dice overlap between single experts and expert consensus [30, 84]. Here, the average Dice accuracy from leave-one-out cross validation will be used—training the algorithm on all but one subject and testing it on the left-out one to compute the algorithm-to-consensus Dice overlap of segmented lesion regions, and iterating until every patient has been left out once and only once.

##### Question 2: Can Z_ADC_ detect lesions in multi-site/scanner/protocol data?

A fundamental problem is that the target patient may have very different distribution of ADC and Z_ADC_ values than the training populations. We will design a self-adaptive mechanism to deal with differences of ADC maps acquired from different sites or scanners. The mechanism will first detect regions of abnormally ADC and Z_ADC_ values in the target patient using information learned from other patients. Then we will re-train the machine classifier on the ADC and Z_ADC_ maps from the target patient, using the tentatively-detected regions as training samples. The assumption is that, the knowledge of lesion voxel appearance as learned from training patients may not be completely suitable for a specific target patient, because of individual differences and sites/scanner/protocol differences. This is especially true for target image voxels that have probabilities of being lesioned just at the border line (e.g., those voxels that computer algorithm thought of having 49% or 51% percent of probabilities being lesioned). On the other hand, the target image voxels that computer algorithms assign very high probabilities of being lesioned (e.g., > 75%) are more reliable. These voxels can serve as “silver standard” to re-train the voxel-wise classifier, using the target image’s features. Re-training using target image voxels for which the tentative results have high confidence to be lesioned or normative voxels can reduce bias in the classifier arising from training on other patients or other imaging protocols, as those “silver standard” voxels are from the same target patient and the same imaging protocol.

### Part3.3. Machine learning of Z_ADC_ and ADC patterns for outcome prediction

#### Part3.3a. Lesion-based outcome prediction

##### Question 3: What are lesion patterns that are associated with neurocognitive outcomes at 2 years of age?

The MRI scoring systems currently used in the hospital setting focus on injuries in certain key brain regions such as the thalamus, basal ganglia, internal capsules, etc. [19, 20, 85]. While the scores reflect the severity of HIE during infancy, the predictive power for outcomes by 2 years of age is not established.

We hypothesize that through machine learning we can find a specific combination of sub-regions with abnormal Z_ADC_ values that better inform outcomes compared to expert scoring systems. One rationale comes from our preliminary results in Fig. 5, which show that injury may involve brain structures not specifically assessed in the current expert scoring systems. In Fig. 5a, we generated probabilistic lesion atlases for HIE. This was based on using our extensively-validated [70] non-rigid registration tool [71] to map individual patients’ lesions into a neonatal atlas [57], where the lesion loads among 141 patients were averaged at the voxel level. For example, a voxel the constructed probabilistic HIE lesion atlas having a value of 0.1 means that 10% of the patients in our cohort had HIE lesions at the same anatomical location. We further used voxel-wise lesion symptom mapping (VLSM [86]) to statistically test whether lesion occurrence in each voxel was significantly associated with treatment (Fig. 5b) and with motor impairment at 2 years (Fig. 5c). The significance was defined as p < 0.05 after 10,000 permutations correcting for potential false positives in multiple comparisons [86]. Panel b shows the lesion atlases in patients who were treated without therapeutic hypothermia (N = 56, mean age at scan = 4 days) and with therapeutic hypothermia (N = 85, mean age at scan = 4 days), and the regions of significant difference between these two cohorts. This quantified the regional reduction in lesion load and shows a larger impact of treatment in the deep gray and posterior structures. Panel c shows atlases in cohorts with (N = 17, mean age 4 days at scan) and without (N = 39, mean age 5 days at scan) clinically-documented motor impairment at 2 years, highlighting that key brain regions associated with adverse motor outcomes involve the anatomic regions mainly along the corticospinal tract (CST) and other cortical structures in occipital and parietal lobes that are not specifically scored in many expert scoring systems.

Our second motivation is that machine learning algorithms can capture subtle patterns that may not be scored in expert scoring systems. For example, two patients may both have injury in thalamus, but may have different outcomes due to slight differences in the volume and location of the injury, in the actual ADC and Z_ADC_ distributions within the injured regions (e.g., whether average Z_ADC_ = − 3 or − 6 in the detected lesion regions as stated under Question 1), in the slight differences in the bi-lateralization, etc. These subtle patterns are not in current expert scoring systems, but can be captured by computer algorithms. In addition, regions that inform outcomes may include both regions with Z_ADC_ < − 2 (abnormally low ADC, possibly ischemic necrosis) as well as regions with Z_ADC_ > 2 (abnormally high ADC, possibly vasogenic edema), but the latter is not considered in expert scoring systems. Table 4 lists all the MRI features (predictive variables extracted from MRI) we propose to use for lesion-based outcome prediction.

**Table 4:** A list features to be used for lesion-based outcome prediction

Categories	Details of features
I. Lesion anatomy	I.1. Mass center in standard neonatal atlas space I.2. Percentage of the whole-brain volume and the volume of each of the 61 auto-segmented brain structures being injured [76, 78, 122] I.3. Ratios of volumetric injury in the same brain structures between the left and right hemisphere I.4. Percentage and distribution of HIE lesions in 28 major fiber tracts as defined in the JHU atlas [123]
II. Lesion geometry	II.1. Lesion volume II.2. Maximum diameter along different orthogonal directions, maximum surface of lesion, lesion compactness, lesion spherecity, surface-to-volume ratio
III. Lesion heterogeneity	III.1. Histogram analysis (0, 25, 50, 75 and 100-percentile) of T1, T2, DWI, ADC, Z_T1_, Z_T2_, Z_DWI_, Z_ADC_ signal values within the lesion regions III.2. Skewness (asymmetry), kurtosis (flatness), uniformity and randomness (entropy and standard deviations) of T1, T2, DWI, ADC, Z_T1_, Z_T2_, Z_DWI_, Z_ADC_ signal values within the lesion regions
IV. Lesion texture	IV.1. gray-level co-occurrence matrix (GLCM) features and gray-level run-length matrix (GLRLM) of T1, T2, DWI, ADC, Z_T1_, Z_T2_, Z_DWI_, Z_ADC_ signal values within lesion regions IV.2. fractal analysis, Minkowski functionals, wavelet transform and Laplacian transforms of Gaussian-filtered images for the lesion regions

#### Part3.3b. Lesion-free outcome prediction

##### Question 4: Can the magnitude and pattern of Z_ADC_ augment outcome prediction?

We hypothesize that outcomes are associated with the magnitude and pattern of Z_ADC_ values. For example, a patient with Z_ADC_ = − 5 in every voxel in a region may have a worse outcome than another patient with values ranging between − 5 and − 2 in the same region.

To test this hypothesis, we will include textures of Z_ADC_ in the multivariate predictive model. The texture will include the entropy, skewness, kurtosis, and histogram analysis (0, 25, 50, 75, 100-percentile) of ADC and Z_ADC_ values in the injured regions. Each patient will be represented as a high-dimensional feature vector. Classification models (e.g., support vector machine (SVM) [87] or random forest (RF) [88]) will be explored for their ability to predict categorical outcomes, and regression models (e.g., support or relevance vector regression (SVR, RVR) [89]) to predict the continuous outcomes [29].

To reduce the risk of over-fitting, our iterative forward inclusion and backward elimination (FIBE) feature selection algorithm will be used [71, 90] to select the most informative subset of features. The FIBE feature selection algorithm will start from the single most informative feature (one with the smallest prediction error in the training set), and iteratively add one feature into the subset at a time, such as adding this feature leads to the maximum decrease of prediction errors with Support Vector Regression compared to adding any other feature, until no features can be added that further reduces the prediction error. The algorithm will then exclude features from the subset, one at a time, such that removing this feature leads to the maximum decrease of prediction errors compared to removing any other feature, until no other features can be removed from the subset that further reduces prediction error. The algorithm iterates between forward inclusion and backward elimination until no feature can be added or remove. The final subset is the selected subset of features that leads to the minimum prediction error. The FIBE algorithm does not start from the full feature set. At any time, the subset only contains a small fraction of all features, therefore it reduces the risk for overfitting.

One merit of using texture features of ADC and Z_ADC_ maps is that we may be able to predict outcomes in ADC maps that are read as clinically normal. It is well known that 30–50% of patients affected by HIE do not show visually explicit lesions [24, 25], which may be due to mild injury, pseudo-normalization (ADC values returning to normal, hiding the lesions before the lesion actually resolves [52]), the use of TH, or other reasons. In these scenarios, voxels may not survive the threshold of Z_ADC_ map by − 2 (since lesions are invisible), but subtle pattern abnormalities can still be captured in the texture of ADC and Z_ADC_ and these patterns may contribute to outcome prediction. Table 5 lists all the MRI features (predictive variables extracted from MRI) that we plan to use for lesion-free outcome prediction. No explicit lesion detection is needed.

**Table 5:** A list features to be used for lesion-free outcome prediction

Categories	Details of features
I. Fiber tract features	I.1. Histogram analysis (0, 25, 50, 75 and 100-percentile) of T1, T2, DWI, ADC, Z_T1_, Z_T2_, Z_DWI_, Z_ADC_ signal values within each of the 28 major fiber bundles as defined in the JHU atlas [123] I.2. Skewness (asymmetry), kurtosis (flatness), uniformity and randomness (entropy and standard deviations) of T1, T2, DWI, ADC, Z_T1_, Z_T2_, Z_DWI_, Z_ADC_ signal values in each brain structures
II. Regional anatomy features	II.1. Histogram analysis (0, 25, 50, 75 and 100-percentile) of T1, T2, DWI, ADC, Z_T1_, Z_T2_, Z_DWI_, Z_ADC_ signal values within the brain and each of the 61 auto-segmented brain structures/regions II.2. Skewness (asymmetry), kurtosis (flatness), uniformity and randomness (entropy) of T1, T2, DWI, ADC, Z_T1_, Z_T2_, Z_DWI_, Z_ADC_ signal values in the brain and 61 auto-segmented regions II.3. Volume of the 61 auto-segmented structures/regions as measured in T1 image II.4. Left/right asymmetry in features II.1–II.3

#### For both Part3.3a and Part3.3b

##### Question 5: Generality to multi-site data?

We will test the hypothesis that certain predictive variables that are more robust to multi-site data will achieve more stable prediction accuracy. For example, lesion volume might be a more stable variable than the boundary irregularity, and the standard deviation of ADC and Z_ADC_ values should be more stable than the actual mean or median values. We will rank variables and variable combinations by their predictive power in data across sites and encourage the auto-selection of stable variable combinations for multi-site generalization.

##### Question 6. Can clinical variables augment MRI metrics and further improve outcome prediction?

We will test this by combining the MRI metrics mentioned above with clinical variables, such as: EEG [91], 1- and 5-min APGAR scores [92], umbilical cord arterial pH value [93], length of stay in neonatal intensive care unit (NICU) [94], as listed in Part 1.3. The combination of MRI and clinical variables will increase the length of the patient-wise feature vector. However, the same feature selection (prediction and accuracy assessment) will still apply.

*Accuracy evaluation* For predicting binary outcomes, we will measure accuracy by sensitivity and specificity in leave-one-out cross validations. That is, we will divide N patients into a cohort of (N − 1) training patients and 1 testing patient. We learn the MRI signatures from known outcomes in the training patients, apply the learned model to predict outcome in the testing patient, and then check whether the predicted outcome for the testing patient is correct or false compared to the actual outcome of this testing patient. We iterate this process N times, such that every patient has been left out once and only once as the testing patient. Sensitivity is measured as the percentage of patients who had adverse outcomes has been correctly predicted, and specificity is measured as the percentage of patients who had normal outcomes has been correctly predicted. Similarly, accuracy for predicting continuously-valued outcomes (Bayley scores) will be measured via the root mean squared error (RMSE) between the predicted and actual scores in cross validations. That is, in a leave-one-out cross validations, ML learns the predictive model from (N − 1) training patients and predicts the Bayley score of a testing patient. We will repeat this N times such that each patient has been left out once and only once as the testing patient. The average RMSE between the predicted and the actual Bayley scores will be used to quantify prediction accuracy. A smaller RMSE means a higher accuracy in prediction.

We will evaluate the accuracies mentioned above for different strategies (lesion-based and lesion-free outcome prediction), for different classifiers during binary outcome prediction (for comparing the accuracies of different classifiers such as SVM, RF, etc.), and for different regressors in during continuous-valued outcome prediction (for comparing the accuracies among regressors such as SVR, RVR, etc.).

*Comparison with expert scoring systems* We will quantitatively compare our Z_ADC_-based predictive model with expert scores, in terms of the accuracy in predicting outcomes in a k-fold or leave-one-out cross validation. Expert scores will be independently determined by 2 pediatric neuroradiologists, using the NICHD–NRN scoring criteria [19].

### Expected sample size and power analysis

The expected sample size is 300 patients with a complete set of MRI and clinical variables, including outcomes. Approximately 440 cases of neonatal HIE cases with accompanying MRI scans have been identified from patients treated in MGH and BCH during 2009–2019 thus far, and our ongoing expert review of clinical records has found that roughly 50–60% of them had outcome data. MGH and BCH admit ~ 50–60 HIE patients annually.

When developing Z_ADC_-based thresholding and machine learning to detect HIE lesions, we will quantify the Dice overlap, sensitivity and specificity of the detected lesion with regard to expert consensus in the leave-one-out manner (under Question 1). Recent machine learning driven HIE lesion detection has a median algorithm-and-expert Dice overlap at 0.52 and from 20 HIE patients (2017) [68]. We can loosely consider their mean Dice as 0.52 (actually not reported [68]). Given a desired power of 0.8, and alpha = 0.05, and assuming the standard deviation of Dice overlaps at 0.15 (which is the case in our pilot data [72]), we need 56 (or 13) patients to say our algorithm has achieved a significantly higher Dice accuracy if our mean Dice is 0.6 (or 0.69 as in our pilot data [72]).

When correlating each MRI metric with outcome scores. We need 46 or 71 subjects to test whether two variables (MRI and outcome) are significantly correlated with a power at 0.95 (beta = 0.05) and 0.995 (beta = 0.005) respectively (|PCC| > 0.5, p < 0.05, where PCC is Pearson’s Correlation Coefficient).

In developing multi-variate prediction models, the one-in-ten-rule [95, 96] states a minimum risk of over-fitting if the algorithm selects, from the anticipated cohort of 300 patients, no more than 30 features that jointly predict outcomes. This is often the case in our similar studies and can be strictly enforced by our feature selection tool [35, 42, 43, 90].

## Discussion

This protocol describes our plans to build on our Z_ADC_ measurement, and to develop machine learning tools to detect lesions and predict outcomes for neonatal HIE patients.

Secondary use of hospital-hosted data has received increasing attention [54–56]. Recent years have seen a harmonization of public informatics platforms that facilitate the mining of clinical databases for big data research [97]. Retrospective data collection is possible because of the now mature hospital data registries and the dissemination of clinical informatics tools. NICU registries record comprehensive clinical information on infant patients. Complementary to registry data, big data search engines allow us to query hundreds of thousands of patients by ICD codes and keywords [98]. Tools for this purpose include i2b2 [99], SHRINE [100], HiGHmed [101], tranSMART [102], etc.; many of which are being adopted in healthcare settings through the world [63–65]. Publicly available platforms such as mi2b2 [66] and ChRIS [103, 104] permit the download of MRI data from Radiology archives with patient MRNs obtained from registry or hospital-wide searches [66]. Experts further filter cases for eligibility and quality control, and review clinical records to ascertain clinical information (e.g. entering outcomes into the REDCap database) [105]. The registries in hospital departments and the public availability of hospital-wide search tools foster data collection that is reproducible among hospitals.

A typical clinical trial often requires significant funding and years to collect data. In contrast, we plan to use existing clinical database. With 1–2 years of effort and limited resources we have identified 440 candidates (141 from MGH and 299 from BCH), the equivalent of 10+ years of active enrollment at the two hospitals. While not all of these candidate patients have a full spectrum of data, the process demonstrates the feasibility of the proposed study to aggregate the necessary cohort from our two hospitals. In contrast, the “Whole Brain Cooling” trial collected data from 208 HIE patients over a 3–4 year time frame (2000–2003) across 16 sites [5, 106–108], the “Optimizing Cooling” trial collected data from 364 HIE patients over 6–7 years (2010–2016) from 19 sites [22, 109–111], the “Late Hypothermia” trial collected data from 168 HIE patients over 8–9 years (2008–2016) from 22 sites [112], and the BABY BAC II trial is aiming to collect data for 160 HIE patients over 3–4 years (2017–2020) from 12 sites [113]. The time and resources saved in data collection makes our protocol a useful complement to existing clinical trials.

Of specific utility to HIE investigations, we recently developed the first-of-its-kind normative pediatric ADC atlases. This valuable resource allows for the quantification of the deviation from normal at the voxel level. We will develop machine learning tools to fully explore and test this new MRI measurement (voxel-wise Z_ADC_) in lesion detection and outcome prediction. This will supplement the interpretation of neonatal brain MRIs, which is currently a subjective assessment performed by experts, by adding data that is quantitative, objective, consistent, anatomically-created and generalizable across multiple sites (Fig. 4). Once validated, the novel and machine-learning-powered tools that build on our new Z_ADC_ measurement will pave the way for future preclinical trials involving patients with HIE (Fig. 1).

Another novelty in the study is to use statistically-rigorous lesion-symptom mapping to quantify key neural substrate for treatment and outcomes (Fig. 5). We do note that the results in Fig. 5 are preliminary (N = 141) and will be updated when more retrospective data is gathered (N = 300 as planned). The update is needed in at least two aspects. The first is to have a larger sample size ideally more balanced between sub-cohorts. Right now, N = 17 patients with and N = 39 without motor impairment (not big number, and imbalanced) had led to findings of the red regions (right column of Fig. 5c) not fully within known motor tracts. The second improvement when sample size is bigger is to purify the data. Neurocognitive outcomes at ~ 2 years are often multifactorial, including impairment in multiple sub-domains that exceed motor and include hearing, visual, memory, development delay, etc. We will start from sub-cohorts with the overall normative versus adverse outcomes. When it comes to sub-domains, simply stratifying them into with and without impairment in one specific sub-domain function may be contaminated but comorbidities. For example, Fig. 5c the red regions in the right column are not all within known motor tracts. We will likely need to use clustering approaches of outcomes to find major branches of multifactorial outcomes, or, to factor out (statistically control for) comorbidities in outcomes. Nevertheless, Fig. 5, especially panels (a) and (b), has shown promise of quantitative and rigorous analysis of lesions and lesion-outcome mapping at the voxel level, which may add knowledge to the current experience-based subjective scoring systems.

Special attention has been paid to the importance of fostering multi-site collaborations and generalizability. We will design specific image analysis algorithms to deal with differences in imaging data from multiple sites (Questions 2 and 5). One example of multi-site/scanner differences is in the diffusion parameters and the number of diffusion directions. An ideal number of diffusion directions is a topic under investigation and some suggested at least 45 directions to construct satisfactory fibers in high-angular-resolution diffusion-weighted imaging (HARDI) [114]. However, existing clinical diffusion MRI protocols in our hospitals used 24–32 diffusion directions. Similar settings are adopted in other hospitals or clinical database for HIE populations [115–117]. One reason is that the purpose is not to construct high angular resolution fibers, but to only create ADC and FA maps for clinical neuroradiology interpretation [24, 60]. For another reason, not having more directions in clinical settings is not to extend MRI scan time on neonates (non-sedation) [24, 60]. The purpose of our study is to retrospectively gather data that has been acquired clinically. So, we cannot change the clinical imaging protocol for neonates. Nevertheless, we plan to record the accuracies of lesion detection and outcome prediction as a function of different sites/scanners, different imaging parameters (b values, number of diffusion directions, etc.). This will provide new and quantitative evidence for future search of an optimal imaging protocol that balances between the clinical considerations (scan time especially for non-sedated neonates, sufficiency for neuroradiology reads of ADC/FA maps, etc.) and higher quality and accuracy of diffusion tensor reconstruction.

When it comes to multi-site data, harmonizing diffusion protocols or images has received increasing interest, especially for normative data [118, 119]. Our study is to retrospectively gather existing data from clinical databases from multiple sites. As the data is from patients, and lesions can appear at varying locations and sizes in the patient data, we used an alternative approach to deal with multi-site data differences. The approach is to design an adaptive lesion segmentation algorithm (see Question 2), which first learns the appearances of lesion voxels from other patients, and re-trains itself using the target patient’s own image voxels that have been deem highly probable to be lesioned or normative. The re-training phase is directly on the target patient, not on training patients which may be scanned in a different site. We will test whether this improves the lesion detection accuracy for multi-site data.

Future larger-scale collaborations and pre-clinical trials across more sites will need to address the limitations of this current protocol. These limitations include: sample size, the need to further test multi-site compatibility, retrospective versus prospective suitability, dealing with variability in treatment guidelines and protocols, and standardization of outcome definitions across institution. In addition, we recognize the need for a secure and stable data warehouse, free release and dissemination of the planned algorithms and software tools; inclusion of additional MRI sequences (e.g., spectroscopy [120, 121]); the exploration and incorporation of other clinical [9–11], biochemical [9–12], and serum [12, 13] biomarkers; and so on. Nevertheless, the current protocol is a novel and needed approach that will provide a basis for larger-scale, multi-site studies.

In summary, this paper describes a registry- and informatics-driven clinical dataset collection protocol to power next-generation machine-learning-based MRI analytics for HIE lesion detection and outcome prediction. This study should benefit HIE clinical trials that incorporate brain MRI. The same technical framework can be used for data collection and biomarker development in other pediatric and adult cohorts, such as those with stroke, tumor and other non-brain disorders.

## Data Availability

The data that support the findings of this study are not yet publicly available, as these methods are continuing to be validated. This group is open to collaboration for future analyses on request from the corresponding authors [YO, PEG].

## Abbreviations

MRI: Magnetic Resonance Imaging
HIE: hypoxic ischemic encephalopathy
TH: therapeutic hypothermia
NICU: neonatal intensive care unit
ADC: Apparent Diffusion Coefficient
MRN: medical record number
ICD: International Classification of Diseases
EHR: electronic health record
BSID-III: Bayley Scale of Infant Development, Version III
NICHD–NRN: National Institute of Children Health and Human Development, Neonatal Research Network
BGT: basal ganglia thalamus
ALIC: anterior limb of internal capsule
PLIC: posterior limb of internal capsule
WS: watershed
Stdev: standard deviation
ML: machine learning
ROC: receiver-operating-curve
VLSM: voxel-wise lesion symptom mapping
CST: corticospinal tract
FIBE: forward inclusion and backward elimination
RMSE: root mean squared error
